# Selective dorsal rhizotomy from indication to rehabilitation: a worldwide survey

**DOI:** 10.1007/s00381-025-06786-5

**Published:** 2025-03-17

**Authors:** Liza M. M. van Dijk, K. Mariam Slot, Tom F. Novacheck, Annemieke I. Buizer, Nelleke G. Langerak, SDR working group, Kristian Aquilina, Xiao Bo, Johannes M. N. Enslin, Jennifer Lewis, Robert P. Lamberts, Nobuhito Morota, Pam Thomason, Simon P. Paget, Marcia E. Ward, Neil Wimalasundera, Meredith Wynter

**Affiliations:** 1 Department of Rehabilitation Medicine, Amsterdam UMC Location Vrije Universiteit Amsterdam, Amsterdam, The Netherlands; 2 Rehabilitation and Development, Amsterdam Movement Sciences, Amsterdam, The Netherlands; 3 Department of Neurosurgery, Amsterdam UMC Location University Van Amsterdam, Amsterdam, The Netherlands; 4 Emma Children’s Hospital, Amsterdam, The Netherlands; 5 Gillette Children’S Specialty Healthcare, Saint Paul, MN USA; 6 Neuroscience Institute and Division of Neurosurgery, Department of Surgery, Faculty of Health Sciences, University of Cape Town, Cape Town, South Africa; 7 Department of Research, Sint Maartenskliniek, Nijmegen, The Netherlands

**Keywords:** Cerebral palsy, Rhizotomy, Selection, Neurosurgery, Rehabilitation

## Abstract

**Purpose:**

Selective dorsal rhizotomy (SDR) is a neurosurgical treatment used worldwide to reduce spasticity. The procedure has undergone many changes since its introduction in the early 1900s, and currently, different centers vary in many aspects of the procedure. We surveyed centers on different continents regarding SDR indications, surgical techniques, and postoperative rehabilitation.

**Methods:**

Ten centers worldwide with SDR experience participated in an online survey preparing for a pre-conference workshop in 2022. The main topics were patient characteristics, the selection process, surgery, and rehabilitation.

**Results:**

Universal suitable candidates for SDR were patients with bilateral spastic cerebral palsy, Gross Motor Function Classification System levels II or III, ages 5 to 7 years, and adequate strength, motor control, and access to postoperative rehabilitation. Centers differed in additional inclusion and exclusion criteria and the use of diagnostic tools. Both single- and multilevel approaches were used, with electrophysiological monitoring applied in all approaches. Intensive rehabilitation was recommended after surgery, followed by a less intensive program, with variations in duration, therapy frequency, modalities used, and follow-up periods.

**Conclusion:**

This survey demonstrated many similarities in several aspects of the SDR procedure in centers performing SDR worldwide, while considerable variability was also seen. The results emphasize the need for standardized reporting of SDR procedures and outcome measures to enable international comparative studies. A Delphi procedure could be a first step to reaching a consensus on outcome measurements, which may lead to a consensus regarding the most suitable candidates, surgical techniques, and rehabilitation programs to improve functional outcomes.

**Supplementary Information:**

The online version contains supplementary material available at 10.1007/s00381-025-06786-5.

## Introduction

Cerebral palsy (CP) is a lifelong neurodevelopmental condition arising from non-progressive disorders occurring in the fetal or infant brain. CP often presents with spasticity, which impairs motor function and may lead to contractures and deformities [1]. Surgical procedures are proposed when non-surgical treatments (e.g., physical therapy, oral antispasmodics, botulinum toxin muscle injections) do not suffice to control spasticity or cause intolerable side effects. There are various surgical procedures to reducing spasticity, with two well-known options being intrathecal baclofen therapy and selective dorsal rhizotomy (SDR) [2].

SDR is an effective treatment option for spasticity reduction in children with CP following strict selection criteria [2]. It also has the potential to change the natural disorder trajectory by preventing growth-related complications, such as contractures and deformities. Dorsal rhizotomies have been reported since the early 1900s [3] and gained popularity after it was modified by Peacock in Cape Town in the early 1980s [4]. In this invasive, irreversible procedure, lumbosacral dorsal rootlets are selectively cut to decrease spasticity.

Over the years, the SDR procedure has undergone several changes, as described by Enslin and colleagues [3]. Peacock introduced significant changes to the dorsal rhizotomy procedure to protect bladder and bowel function. He switched from a conus-level approach to a multi-level laminectomy of L2 to L5, followed by an SDR at the level of the cauda equina (L2-S1). He performed selective dorsal fascicular sections based on clinical spasticity patterns and electromyography (EMG) findings, using the principles of the interpretation of EMG as described by Fasano [5]. Later, Park modified the technique, including a single-level laminectomy approach, using ultrasound to localize the conus medullaris to determine which lamina to remove (usually L1 or L2), and performed SDR on L1-S2 at the conus level [6]. Advances in intra-operative neuromonitoring played a central role in improving the SDR procedure, as the pudendal plexus could be much better protected. Today, medical centers perform both the cauda equina and the conus-level approach, with some centers performing a laminoplasty and others a laminectomy.

SDR has been accepted as a “to-do intervention” (green light) for reducing spasticity in children with CP [2]. However, there are also concerns about the long-term effects on functional outcomes [7–9], and a prevalence of 19% post-surgery complications has been reported [10]. This ensures continued development in the SDR procedures.

Currently, there is variation in SDR procedures, including differences in the selection criteria, surgical techniques, and postoperative rehabilitation. This study aimed to provide an overview of SDR regarding indication, surgical approach, and rehabilitation in centers performing SDR worldwide to create more insight.

## Methods

In preparation for a pre-conference workshop in 2022, an online document was sent to centers with expertise in performing SDR. Attention was paid to continental diversity and diverse professional backgrounds (e.g., neurosurgeons, rehabilitation physicians, physiotherapists) without presuming to have approached all global centers of expertise. The experts were asked to provide data regarding: (i) patient characteristics (diagnosis, Gross Motor Function Classification System (GMFCS), age), (ii) selection process (assessors, methods, inclusion and exclusion criteria), (iii) surgery (surgical technique, intraoperative neuromonitoring), (iv) rehabilitation process (inpatient/outpatient, frequency, orthotics, modalities), and (v) postoperative evaluation (follow-up and assessments). The blank version of the document can be found in Supplementary Information 1.

Ten centers where SDRs were performed regularly, based on five continents, participated in an international workshop: two from North America, two from Asia, two from Europe, one from Africa, and three from Australia. The three Australian centers jointly provided the Australian data as they all follow similar clinical practices regarding SDR. Therefore, their data were presented collectively as one center, describing the outcomes of eight centers in total. Details of the participating centers can be found in Supplementary Information 2.

## Results

### Patient characteristics

**Fig. 1:**
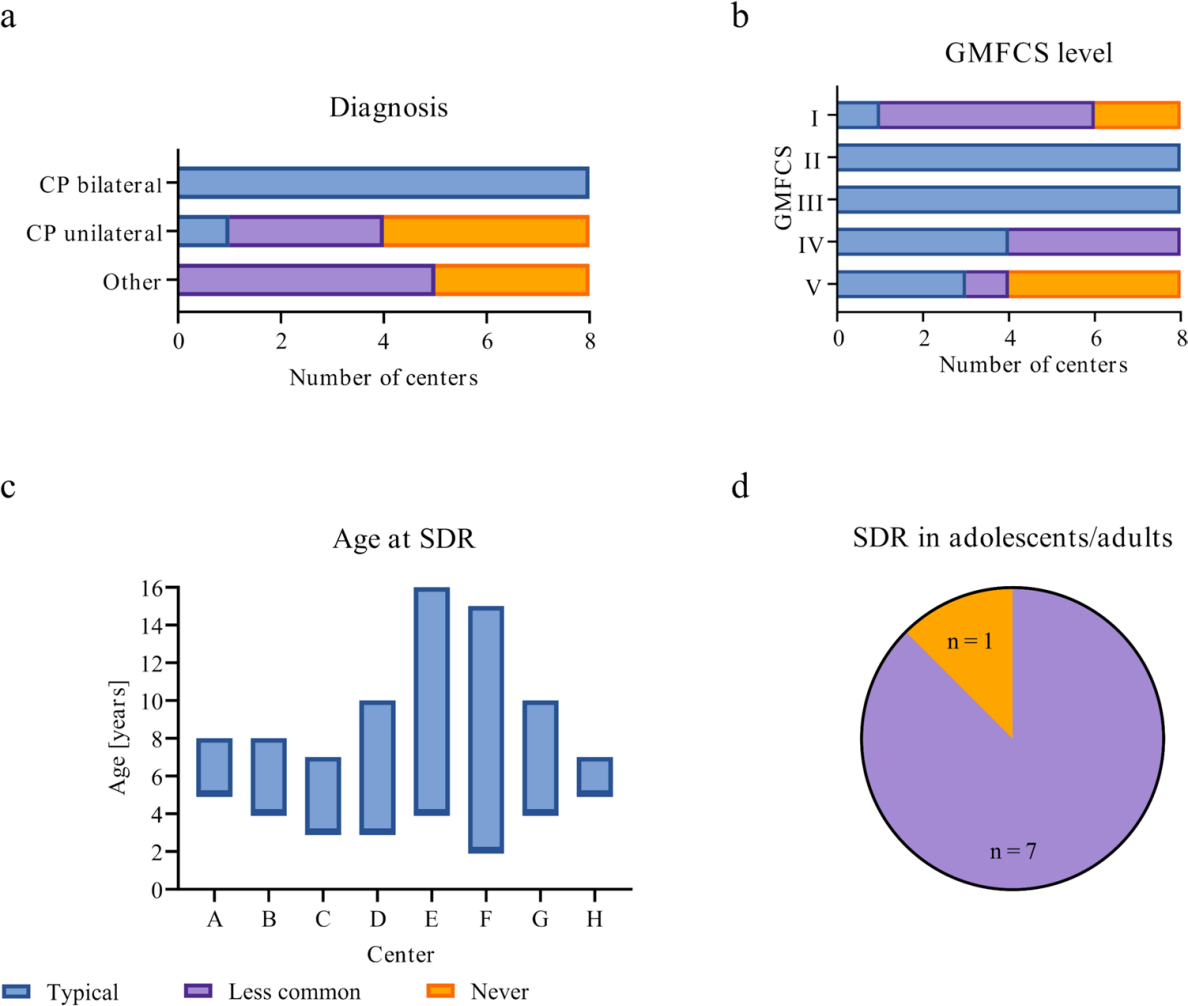
Charts depicting patient characteristics at which centers perform SDRs: **a** diagnosis, **b** GMFCS classification, **c** typical age range, and **d** performance of SDRs in adolescent/adult patients. CP, cerebral palsy; GMFCS, Gross Motor Function Classification System; SDR, selective dorsal rhizotomy

The results regarding patient characteristics are summarized in Fig. 1. All centers included individuals diagnosed with bilateral spastic CP (Fig. 1a), classified with GMFCS level II and III (Fig. 1b), and aged between 5 and 7 years old (Fig. 1c). Over half of the centers conducted SDRs also in individuals with conditions other than CP (Fig. 1a). Three centers frequently operated on individuals with GMFCS level V, while four centers never did (Fig. 1b). The typical age range for SDR procedures varied from 2 to 16 years (Fig. 1c). With the exception of one center, all occasionally performed SDRs on adolescents or adults (Fig. 1d).

### Selection procedure

**Fig. 2:**
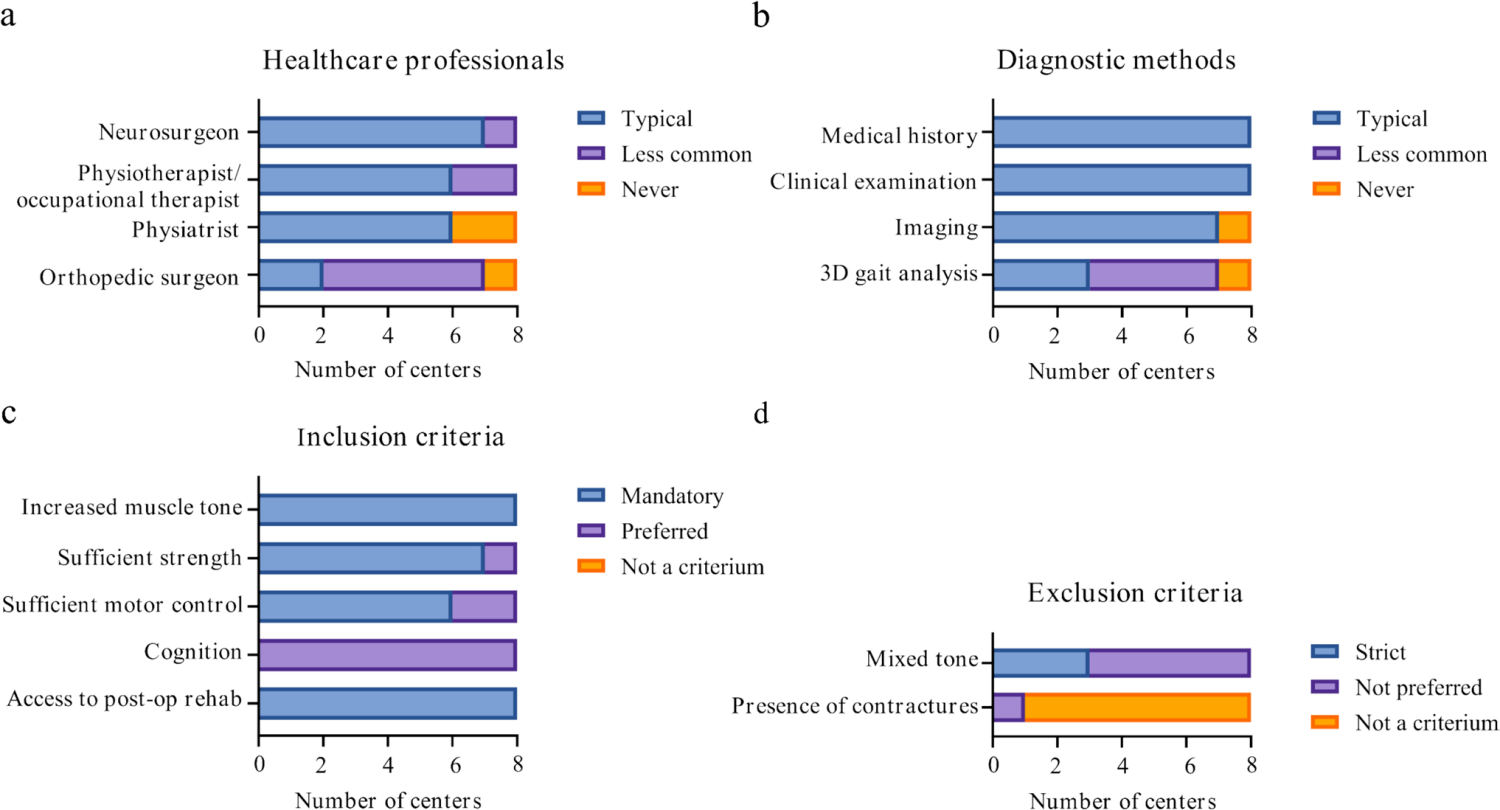
Charts depicting information about the selection procedure: **a** healthcare professionals involved in the selection procedure, **b** diagnostic methods included in the selection procedure, **c** inclusion criteria, and **d** exclusion criteria

Information about the selection process is shown in Fig. 2. In all centers, suitable candidates were selected by a multidisciplinary team involving various specialists and allied health professionals (Fig. 2a). In addition to the medics shown in Fig. 2a, in four centers, the pediatric neurologist was also part of the multidisciplinary team, and in one center also the psychologist, social worker and movement analyst were involved. Suitable candidate selection was based on medical history and physical examination in all centers (Fig. 2b). Nearly all centers also used imaging and 3D gait analysis. Other diagnostic methods used were treatment with botulinum toxin injections and genetic testing if imaging showed no brain or spinal cord abnormalities. The universal inclusion criteria encompassed spasticity, adequate strength, adequate motor control, and access to postoperative rehabilitation (Fig. 2c). Other inclusion criteria mentioned included parental expectations, motivation, and the family’s capacity to participate in rehabilitation and follow-up. All centers assessed patients for the presence of mixed tone. In three centers, mixed tone was a reason for exclusion (Fig. 2d). Other reasons for exclusion were too many prior orthopedic procedures, planned orthopedic interventions, prior muscle lengthening, presence of athetosis, and unrealistic family expectations.

### Surgical technique

**Fig. 3:**
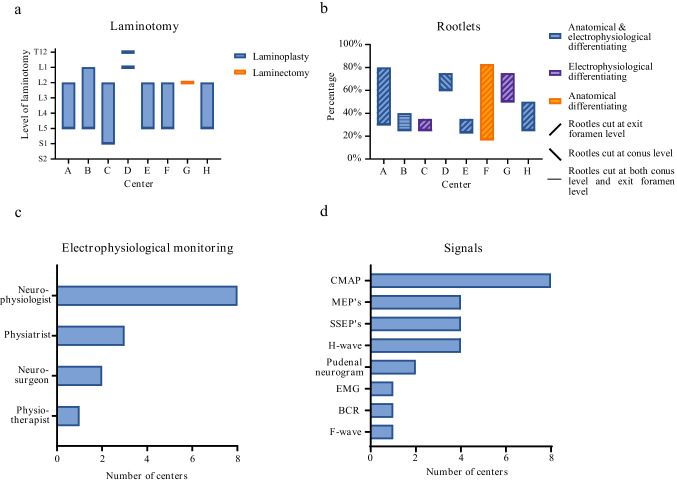
Charts depicting the surgical techniques regarding: **a** the level of laminotomy and if centers perform a laminectomy or laminoplasty, **b** the percentage range and location of rootlets cut and how centers differentiate between motor and sensory rootlets, **c** who is involved in electrophysiological monitoring, and **d** what signals are measured. BCR, bulbocavernosus reflex; CMAP, compound muscle action potential; EMG, electromyography; MEPs, motor evoked potentials; SSEPs, somatosensory evoked potentials

The results regarding the surgical techniques are shown in Fig. 3. Among the eight participating centers, most centers exclusively performed the multi-level approach, some used the single-level approach, and one center performed both multi-level and single-level SDRs. All centers performing the multi-level approach performed the laminotomy on at least laminae L2 to L5 (Fig. 3a). With the exception of one center, all centers conducted laminoplasty (Fig. 3a). The percentage of nerve rootlets transected varied among centers, ranging from 17% (1/6) to 83% (5/6) (Fig. 3b). All centers consistently utilized electrophysiological monitoring during surgery, with continuous monitoring by at least a neurophysiologist, and this process always encompassed the compound muscle action potential (CMAP) monitoring (Fig. 3c, d). The decision of which rootlets were cut was made during surgery in all centers, and depending on the method used (multi- or single-level), it was done at the level of the conus medullaris or at the foramen exit.

### Rehabilitation

**Fig. 4:**
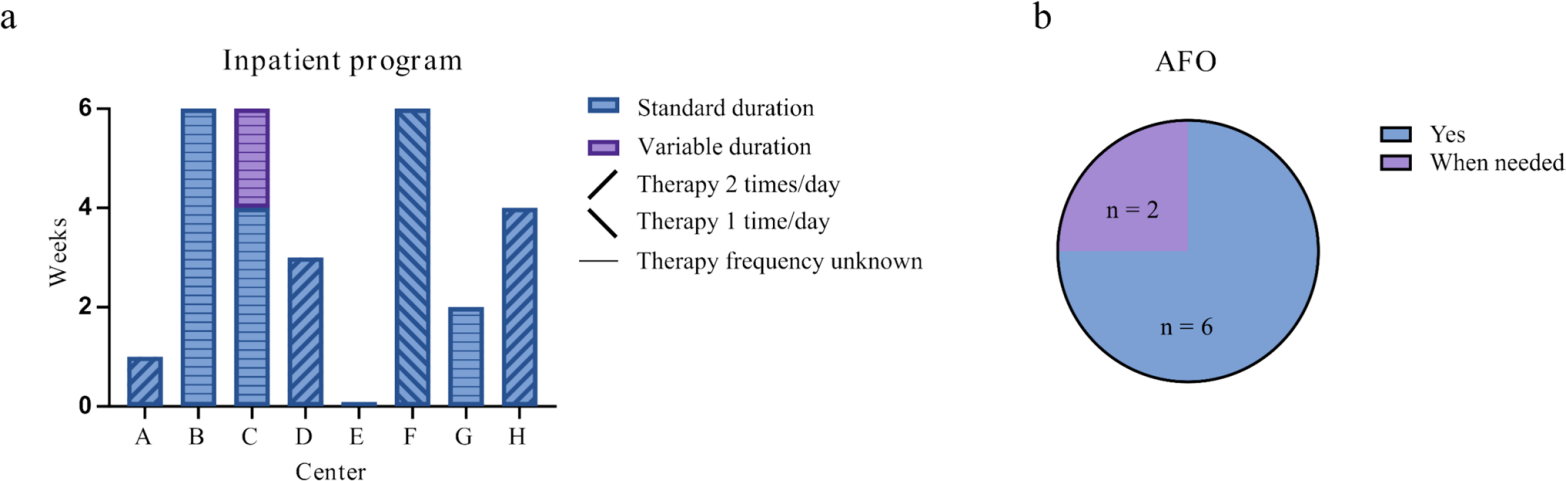
Charts depicting: **a** duration and therapy frequency of the inpatient rehabilitation program, and **b** if ankle foot orthoses (AFO) are prescribed

Information about the rehabilitation program is shown in Fig. 4. The duration and therapy frequency of the inpatient rehabilitation varied among the different centers (from zero to six weeks, with one or two therapy sessions per day) (Fig. 4a). There was also a wide variation in the duration and frequency of the outpatient rehabilitation program, depending on whether a center had an outpatient rehabilitation program or only (a more extended) inpatient rehabilitation program, or vice versa (Supplementary Information 3). Still, all centers had a more intensive rehabilitation program followed by a less intensive program. The modalities used depended on what was available in the particular center, and most centers prescribed ankle foot orthoses (AFOs) (Fig. 4b). All centers had a follow-up with their patients postoperatively. The follow-up period varied, but the majority had a follow-up appointment scheduled at 6 months and one year postoperatively and then followed their patients annually. Detailed information on the rehabilitation process can be found in Supplementary Information 3.

## Discussion

To our knowledge, this is the first comprehensive cross-sectional survey that attempts to provide an overview of the indication for SDR, surgical details, and the postoperative rehabilitation procedure from centers around the world. Although centers performing SDRs share many similarities in several facets of the procedure, there is also considerable variability.

### Selection criteria

SDR is widely acknowledged as a viable treatment option for reducing spasticity to improve function [2, 11, 12]. Selecting suitable candidates is important and is usually guided by a multidisciplinary team, although with varied team compositions. In all participating centers, SDR was performed in children with bilateral spastic CP, classified in GMFCS levels II and III. The age range in which participating centers generally performed SDR varied, spanning from 2 to 5 years at the minimum and 7 to 16 years at the maximum. Even though only minor differences were found in minimum age, this age span is important for gains in gross motor function and ability to cooperate with testing and rehabilitation. While some evidence implies more benefit from SDR in younger patients, positive outcomes have also been described in older children and adults [13].

Interestingly, beyond CP, over half of the participating centers also performed SDR for other etiologies, with hereditary spastic paraplegia (HSP) and post-traumatic spasticity being among the most frequently mentioned diagnoses in children in the sparse literature [14–17]. The short- to medium-term safety and viability of SDR in genetically confirmed HSP cases may be promising in carefully selected patients [16, 17]. Additionally, the use of SDR for patients with post-traumatic spasticity appears to be acceptable [14].

Additionally, there was a notable difference in the consideration of SDR for non-ambulant children (GMFCS levels IV and V) among centers. Since the approval of intrathecal baclofen therapy by the FDA in 1992 [18], most non-ambulatory children with CP were treated with intrathecal baclofen, reserving SDR for ambulant children to improve their gait function [19]. However, growing evidence suggests that SDR may benefit these non-ambulant patients, albeit with a shifted focus towards enhancing daily care and improving comfort rather than gait improvement [19]. Nonetheless, the extent of the impact on health-related quality of life for these patients remains inadequately understood [19]. Delving into SDR for these non-ambulant patients is not only interesting but also necessary to enhance our understanding of the impact of SDR, which could be achieved by defining uniform and standardized outcome measures.

Nearly all centers incorporated imaging for diagnostic purposes, yet less than half used its results for patient selection. MRI evaluation before SDR seems essential for assessing the extent of brain injury, excluding other causes of spasticity and upper motor neuron pathology, and may help predicting SDR outcome [20, 21]. Children without brain abnormalities or with periventricular leukomalacia tend to improve after SDR [20–22]. In contrast, those with significant brain damage in other areas, such as basal ganglia, face a less favorable prognosis [20–22]. In these cases, (masked) dystonia is also regularly present, which is often grounds for exclusion of SDR [23, 24]. Hence, identifying possible complications preceding SDR is significant and, contingent upon the center’s capabilities, conducting a standard MRI before SDR for diagnostic purposes could facilitate this process.

Gait analysis is another valuable diagnostic tool for gaining insight into potential underlying impairments causing gait abnormalities. It aids diagnostic thinking and treatment planning [25]. As the primary goal of SDR in ambulatory pediatric patients is to improve and maintain gait function [24, 26], it may be valuable to perform a gait analysis before SDR to assess the gait status before and after intervention and rehabilitation. Three centers reported routine 3D gait analysis, but alternative gait analysis approaches may be utilized by the other centers, depending on their capabilities. Besides assessing the spastic gait pattern, the presence of contractures and their impact on gait abnormalities seems essential, as research has demonstrated that ambulant patients with contractures derive the least benefit from SDR [4]. Despite this evidence, most surveyed centers did not consider contractures as one of the selection criteria, but some identified the contractures and treated them as adjuvant care.

### Surgical technique

Multi-level and single-level surgical approaches are both performed. This survey only inquired some information regarding surgical technique: multi-level or single-level, the number of laminae involved, and whether a laminectomy or laminoplasty was performed. It did not collect more detailed information about the specific technique used (cauda equina exposure or cone exposure), which could provide a more accurate understanding. Labeling just multi-level or single-level techniques implies two groups of techniques, whereas the reality is more nuanced. Additionally, the term single-level can sometimes be misleading, as the conus medullaris may be positioned unfavorably, requiring the removal of two laminae rather than just one. Therefore, some experts argue that referring to the techniques as conus-level and cauda-level instead of multi-level and single-level techniques would be more appropriate.

The current and limited literature comparing surgical approaches does not favor one of the SDR techniques and showed no apparent difference in short-term functional outcomes, postoperative pain or time to mobilization [27–30]. One study reported a reduced length of stay after the single-level approach [29]. The single-level approach presents a higher degree of technical complexity when compared to the multi-level approach, which has the advantage of making the spinal level of the root precise [6, 29]. Still, single-level surgery has the advantage of less invasiveness of the wound [20]. Therefore, the choice between these approaches often hinges on the prior training and preference of the neurosurgeon and the preference of the patient.

Amidst uncertainty regarding the precise impact of electrophysiological monitoring on SDR outcomes, all centers used it during surgery. Despite the doubts, several studies still recommend its use when the technique is available, as it can help identify which dorsal roots to cut and to what extent [31, 32]. The degree of rootlet division is highly variable among participating centers in this report (17–83%). Even though one study showed a correlation between the sectioned dorsal root percentage and functional improvement [33], near complete elimination of spasticity in long-term follow-up is reported by others [7, 11].

### Rehabilitation

The centers reported that ambulant patients undergo an intensive rehabilitation program, which is essential after an SDR [26]. The prescription of AFOs to gain stability and alignment is often part of standard care [34], as reported in most participating institutes in the survey. Nonetheless, substantial differences exist in the length of inpatient rehabilitation programs and the frequency of therapy sessions during the inpatient stay. The initial intensive phase of the outpatient rehabilitation program is transitioned to a less intensive program in most centers, which could last up to a year after SDR, with annual follow-up appointments.

### Limitations

A limitation of this survey is that only ten centers with expertise in SDR participated. There are many more centers with SDR experience worldwide, so there is some selection bias. Nevertheless, by inviting centers from different continents, an attempt was made to consider cultural differences.

### Future research

Due to high variability in selection criteria, surgical technique, and rehabilitation, interpreting SDR outcomes is complex. Therefore, future research should focus on unifying outcome measurements to enable international comparative studies with real-life data. A Delphi procedure could be a first step to reaching a consensus on standardizing outcome measurements, enabling the comparison of worldwide research more easily. This information could be used to improve selection and surgery methods and ultimately improve functional outcomes.

## Conclusion

This study provides an overview of the indication for SDR, surgical techniques, and postoperative rehabilitation, as applied in ten centers performing SDR localized on five continents. There is a wide variation in all aspects of the SDR procedure, which makes interpreting SDR outcomes challenging. The results of this study emphasize the need for standardized outcome measurements to assess the optimal candidates and treatment strategy. Future research could provide further insight into these uncertainties and may lead to a consensus regarding the most suitable candidates, surgical techniques, and rehabilitation programs to improve functional outcomes.

## Supplementary Information

Below is the link to the electronic supplementary material.

Supplementary file1 (PDF 518 KB)

## Data Availability

No datasets were generated or analysed during the current study.
