# Supplementary material for: Selective dorsal rhizotomy from indication to rehabilitation: a worldwide survey
Source: Childs Nerv Syst. 2025 Mar 17;41(1):133. doi: 10.1007/s00381-025-06786-5 (PMC11913937; doi:10.1007/s00381-025-06786-5)
Supplement: Supplementary file 1 — Supplementary file1 (PDF 518 KB) [file 381_2025_6786_MOESM1_ESM.pdf]

Supplementary Material

Selective dorsal rhizotomy from indication to rehabilitation: A worldwide survey

Journal: Child's Nervous System

Liza M.M. van Dijk[1,2], K. Mariam Slot[3,4], Tom F. Novacheck[5], SDR working group, Annemieke I. Buizer[1,2,4]*, Nelleke G. Langerak[6,7]*

[1] Department of Rehabilitation Medicine, Amsterdam UMC location Vrije Universiteit, Amsterdam, The Netherlands.

[2] Rehabilitation and Development, Amsterdam Movement Sciences, Amsterdam, The Netherlands.

[3] Department of Neurosurgery, Amsterdam UMC Location Universiteit van Amsterdam, Amsterdam, The Netherlands.

[4] Emma Children's Hospital, Amsterdam UMC, Amsterdam, The Netherlands.

[5] Gillette Children's Specialty Healthcare, Saint Paul, MN, USA.

[6] Division of Neurosurgery, Neuroscience Institute, University of Cape Town, Cape Town, South Africa.

[7] Department of Research, Sint Maartenskliniek, Nijmegen, the Netherlands.

Email corresponding author: nellekelangerak@hotmail.com

Supplementary Information 1: Empty tables sent to participating centers

Diagnosis

Center	Diagnosis			GMFCS					Age	
	CP bilateral	CP unilateral	Other	I	II	III	IV	V	Children (typical age range)	Adolescent or Adult

XX = typical / X = will do, but less common / 0 = will not do/essentially never
Abbreviation: GMFCS: CP: Cerebral palsy, Gross Motor Function Classification System

Selection Process

Center	Who is involved in selection process?					Method				
	Neuro-surgeon	Orthopedic surgeon	Physiatrist	PT / OT	Other	History	Examination	Imaging	3D Gait analysis	Other

XX = typical / X = will do, but less common / 0 = will not do/essentially never
Abbreviations: CP: Cerebral palsy, OT: occupational therapist, PT = physiotherapist

Selection criteria

Center	Inclusion Criteria								
	Age (range)	Etiology of CP	Muscle tone	Strength	Motor control	Cognition	CNS imaging	Access post-op rehab	Other

XX = need to be eligible for SDR / X = preferably, not necessary / 0 = not a selection criteria
Abbreviations: CNS: central nervous system, CP: cerebral palsy, post-op rehab: post-operative rehabilitation.

Center	Exclusion Criteria				
	Not CP	Mixed tone	Contractures	No access to post-op rehab	Other (list, e.g. prior surgery)

XX = absolute exclusion criteria for SDR / X = not preferably, but not an absolute exclusion criteria / 0 = not a selection criteria
Abbreviations: CP: cerebral palsy post-op rehab: post-operative rehabilitation.

Surgical technique

Center	Technique	Laminectomies		Use of electro-physiological mapping		
	Multi- or single level	Level of laminotomies (range)	Laminectomy *or* laminoplasty	Mandatory, mostly or never	Monitored: Surgeon, other MD, Neurophysiologist or PT	CMAP, MEP’s, SSEP’s or H-wave

Abbreviations: CMAP: Compound muscle action potential, MD: medical doctor, MEP’s: motor evoked potentials, PT: physiotherapist, SSEP: Somatosensory Evoked Potentials, tEMG: Triggered Electromyography.

Center	Rootlets				
	Decision pre-op or during surgery	Percentage rootlets (range)	Level of cutting rootlets: Conus or at exit foramen	Differentiating motor vs sensory: Anatomical, Electrophysiological or combination	Use of EMG guidance

Abbreviation: EMG: Electromyography.

Rehabilitation

Center	Rehabilitation				
	Time inpatient program (weeks)	Time outpatient program (weeks)	Frequency of therapy (hospital/community)	Use/Type of orthotics	Modalities used (e.g. lokomat, hydrotherapy)

Post-operative evaluation

Center	Follow-up
	Frequency of check-up Post-operative Assessment If so, outcome measures used

Supplementary Information 2 : Participating centers

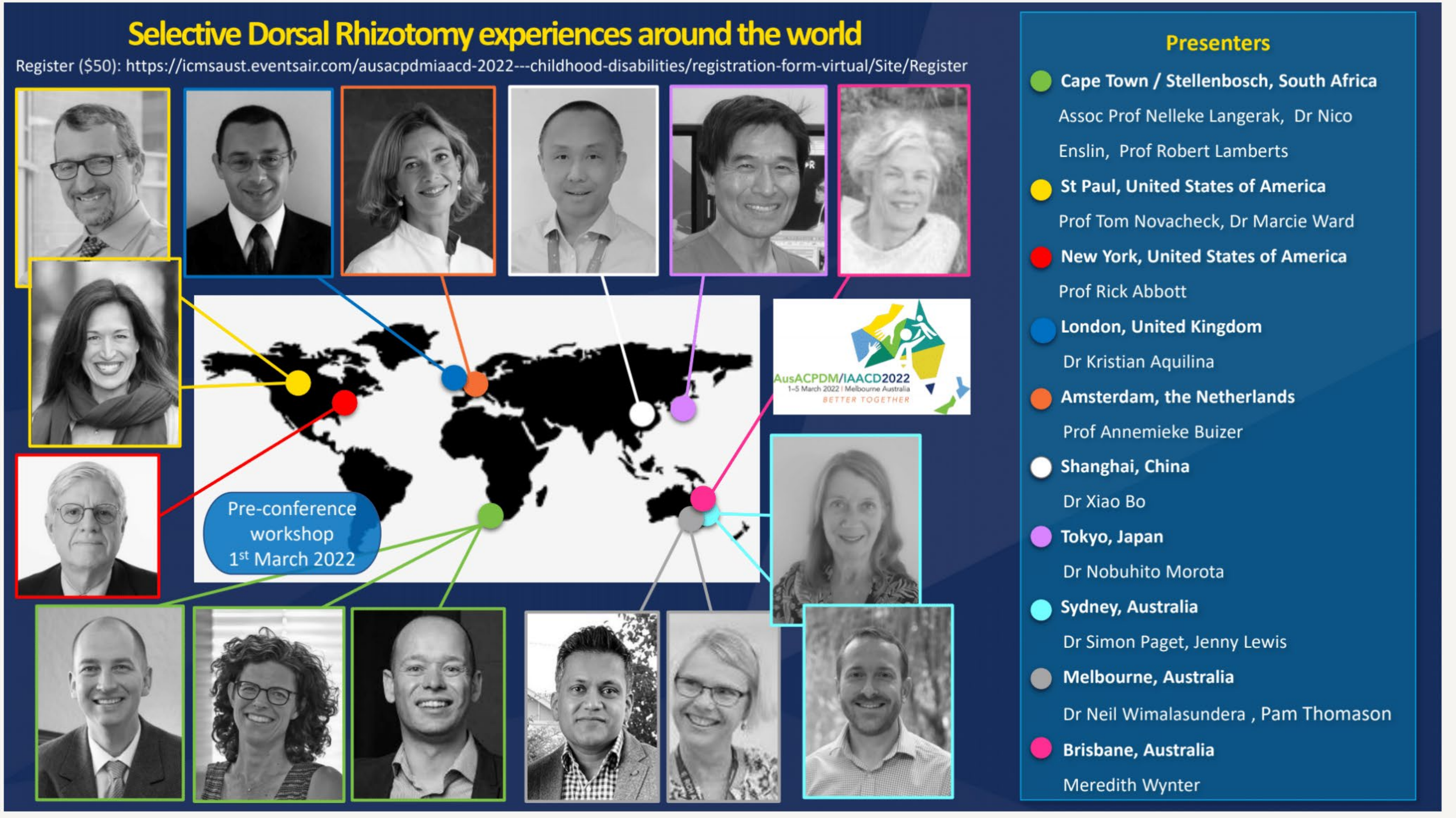

Supplementary Information 3: Filled tables regarding the rehabilitation process

**Center**	**Rehabilitation**				
	Time inpatient program (weeks)	Time outpatient program	Frequency of therapy (hospital/community)	Use/Type of orthotics	Modalities used (e.g. lokomat, hydrotherapy)
**A**	1	Daily for 2 weeks, then 2-3x/week, intense blocks prn	Hospital 2x/day in week 1; home: daily in week 2-3; then 2-3x/week	Only if needed for function (AFO's)	NDT
**B**	6	Daily for 3 months, then 2-3x/week		Stiff PLS AFO	Lokomat for GMFCS III/IV, hydrotherapy, treadmill
**C**	4-6	Daily, then 2-3x/week for 5-6 months	Hospital 3 sessions; home daily 45-60 minutes for 5-6 months, 45-60 minutes 2x/week	AFO with check strap to allow variable dorsiflexion as tolerated	
**D**	1 (hospital ward)	2 weeks (stay in hospital in patient hotel)	Twice daily for 3 weeks; then 3x/week after discharge	AFO	Hydrotherapy, treadmill
**E**	0	5x/week for 3 months, then 2-3x/week up to a year post surgery	Community: physiotherapy 5x/week for 3 months, then 2-3x/week up to a year post surgery	Rigid ventral AFOs for the first year, thereafter, depending on gait parameters	Functional gait training and strength training
**F**	6	None<br>(evaluation 1x/year)	Hospital: 1x/day for 6 weeks; community: 2-4x/month for 12 months; 1-2x/month thereafter; home: 1-3x/day	Whenever needed	None
**G**	1.5	12 months minimal	5 days/week, 6 hours/day	Stiff PLS AFO	Treadmill
**H**	4	2-6 weeks daily-, intensive blocks prn	Hospital: 2x/day for 6 weeks; community: 2-3x/week for 12 months, 2x/week for 12-24 months	SAFOs +/- wedge	Hydrotherapy, treadmill, Litegait, FES, cycling, lokomat

Abbreviations: AFO: Ankle-foot orthosis, FES: Functional electrical stimulation, GMFCS: Gross Motor Function Classification System, NDT: Neurodevelopmental treatment, PLS: Posterior-leaf-spring, Prn: Pro re nata (as needed), SAFO: Silicone ankle-foot orthosis.

Center	Follow-up		
	Frequency of check-up	Post-operative Assessment	If so, outcome measures used
**A**	At 6 months and 12 months, then yearly	Yes	Clinical only – function assessment
**B**	At 6 months, and 12 months, then yearly	Yes	3DGA, MAS, FMS, Gillette FAQ, GMFM-66
**C**	At 3 months, and 6 months, then yearly	Yes	Clinical exam, radiographs as indicated
**D**	At 6 months, 12 months, and 2 years	Yes	MAS, GMFM 66, CP QOL, TUG, 3DGA when appropriate
**E**	First year: at 3-6-9-12 months, then yearly	Yes	3DGA, FMS, GMFM-66, SPAT, functional strength, COPM, CPHILD, Caregiver Questionnaire, X-rays hip and spine
**F**	At 1 month, then every 3 months for a year, then every 6 months for the next 4 years, then yearly	Yes	GMFM-88, MAS, Wee-FIM, Video
**G**	Every 3 months	Yes	MAS, GMFM-66, GMFCS, 3DGA
**H**	Research: Baseline; at 12 months, 2-5-10 years Clinical: + at 3 months, and 6 months, then annually	Yes	Imaging, 3DGA, MAS, MMST, (HAT), FCSs, FMS, Gillette FAQ, 6MWT, TUG, GMFM-66, COPM, CP-QoL, FACES pain, Promis pain interference

Abbreviations: 3DGA: 3D gait analysis, 6MWT: 6-minute walk test, COPM: Canadian Occupational Performance Measure, CPCHILD: Caregiver Priorities and Child Health Index of Life with Disability, CP-QOL: Cerebral Palsy Quality of Life, FCSs: Family Community Support service, FMS: Functional Mobility Scale, Gillette FAQ: Gillette Functional Assessment Questionnaire, GMFCS: Gross Motor Function Classification System, GMFM: Gross Motor Function Measure, HAT: Health Assessment Tool, MAS: Modified Ashworth Scale, MMST: Manual Muscle Strength Test, SPAT: Spasticity Test, TUG: Timed Up and Go Test, Wee-FIM: Functional Independence Measure for children.
